# Wire In Pigtail cathEteR ThRoUgh Snare Twice (WIPER TRUST) technique for grasping leads without free ends in transvenous lead extraction

**DOI:** 10.1016/j.hrcr.2025.07.005

**Published:** 2025-07-11

**Authors:** Yuhei Kasai, Junji Morita, Takayuki Kitai, Takuya Haraguchi, Tsutomu Fujita, Kazuhiro Satomi

**Affiliations:** 1 Department of Cardiology, Asia Medical Group, Sapporo Heart Center, Sapporo Cardiovascular Clinic, Sapporo, Hokkaido, Japan; 2 Department of Cardiology, Tokyo Medical University, Shinjuku, Tokyo, Japan

**Keywords:** Transvenous lead extraction, Tandem approach, 0.014-in guidewire, Pigtail catheter, Lead with inaccessible ends, Wire TRUST technique, Needle’s eye snare

**Key Teaching Points**

• Tandem approach combining superior and femoral routes is a highly effective strategy for transvenous lead extraction (TLE), especially in challenging cases with prolonged lead dwell times. This approach improves coaxial alignment of extraction sheaths and enables dual-sided traction, which enhances dissection control and facilitates safe removal. It also reduces the risk of superior vena cava injury and improves the overall procedural success rate in complex extractions.
• The WIPER TRUST (Wire In Pigtail cathEteR ThRoUgh Snare Twice) technique is an enhanced evolution of the original Wire TRUST (Wire ThRoUgh Snare Twice) technique for grasping leads without free ends during TLE. While the Wire TRUST technique uses only a 0.014-in guidewire, the WIPER TRUST technique adds a 4-F pigtail catheter, significantly improving control, coaxial force application, and lead insulation safety. This modification minimizes the risk of guidewire kinking and insulation embedding, making the procedure safer and more reproducible, particularly in complex extractions.
• The WIPER TRUST technique remains effective even in multiple lead extraction cases, offering procedural flexibility and efficiency. Compared with the needle’s eye snare, which requires complex manipulation and frequent repositioning, the WIPER TRUST technique allows regrasping of multiple leads using a single snare catheter and additional pigtail catheter, reducing both the procedure time and the cost. This approach is particularly advantageous when managing one lead with inaccessible ends and another with accessible ends.

## Introduction

Transvenous lead extraction (TLE) is a cornerstone of long-term management for patients with cardiac implantable electronic devices. Indications for TLE include lead infections, lead malfunction, and the requirement for lead replacement or upgrade to cardiac resynchronization therapy.^1^ The superior approach is commonly used as the first-line strategy for TLE. In complex cases, such as lead extraction with prolonged dwell times, a combined superior and femoral approach may be required to ensure safe and effective removal.^2^ The Wire ThRoUgh Snare Twice (Wire TRUST) technique is effective for grasping leads with inaccessible ends.^3^^,^^4^ In this report, we propose an advanced variation of this technique, called the Wire In Pigtail cathEteR ThRoUgh Snare Twice (WIPER TRUST) technique, for grasping leads without free ends during TLE.

## Case report

A 78-year-old woman with a history of intermittent complete atrioventricular block underwent dual-chamber pacemaker implantation (Accent MRI, Abbott, Chicago, IL) 10 years previously. She was referred to our hospital. A pacemaker evaluation showed multiple episodes of electrical artifacts in the right ventricular (RV) lead (Tendril MRI, 52 cm, Abbott), while the right atrial (RA) lead (Tendril MRI, 46 cm, Abbott) functioned normally. The patient was scheduled for TLE, RV lead replacement, and pacemaker generator exchange.

TLE was performed using a combined superior and femoral (tandem) approach to ensure coaxial alignment of the powered sheath with the RV lead. We first removed the device, and the lead and suture sleeve were exposed using blunt dissection. A locking stylet (LLD EZ, Philips, Andover, MA) was advanced toward the RV lead tip, but the helix could not be unscrewed. We secured new RV lead access via a distal puncture and simultaneously initiated the femoral approach using the WIPER TRUST technique.

**Figure 1:**
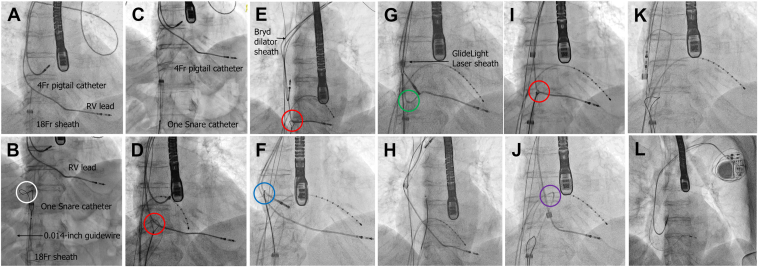
Fluoroscopic images of the procedure. **A:** A 4-F pigtail catheter hooked the ventricular lead under fluoroscopic guidance. **B:** The distal tip of a 0.014-in guidewire was navigated through the ONE Snare (*white circle*). **C:** The ONE Snare catheter, securely grasping the 0.014-in guidewire, was carefully retracted into an 18-F femoral sheath, enabling the externalization of the 0.014-in guidewire together with the 4-F pigtail catheter, which had been firmly secured to the guidewire using a torque device. **D:** The right ventricular (RV) lead was securely grasped by advancing and tightening the snare while applying tension to the externalized 4-F pigtail catheter (*red circle*). **E:** After successfully establishing the Wire In Pigtail cathEteR ThRoUgh Snare Twice (WIPER TRUST) technique via the femoral approach (*red circle*), we performed dissection of adhesion using a mechanical sheath through the superior approach. **F:** The snare catheter was reinserted into the 18-F sheath independently of the pigtail catheter and was used to grasp the free end of the right atrial (RA) lead (*blue circle*). **G and H:** The RA lead was successfully extracted using the 14-F and 16-F GlideLight laser sheaths by applying traction to the proximal (superior approach) and distal (femoral approach via the externalized pigtail catheter [*green circle*]) ends of the RV lead. **I:** After regrasping the RV lead with the ONE Snare and externalized 4-F pigtail catheter, the WIPER TRUST technique was successfully reestablished, allowing repeated dissection of adhesion using the 16-F GlideLight laser sheath via the superior approach. **J:** After completing dissection of adhesion up to the grasping point, the pigtail catheter was gently advanced upward to prevent interference with the superior approach (*purple circle*). **K:** The RV lead was successfully extracted by applying countertraction with the 16-F GlideLight laser sheath. **L:** A new RV lead was implanted in the ventricular mid-septum, and a new RA lead was positioned in the RA appendage; both were connected to a new generator.

**Figure 2:**
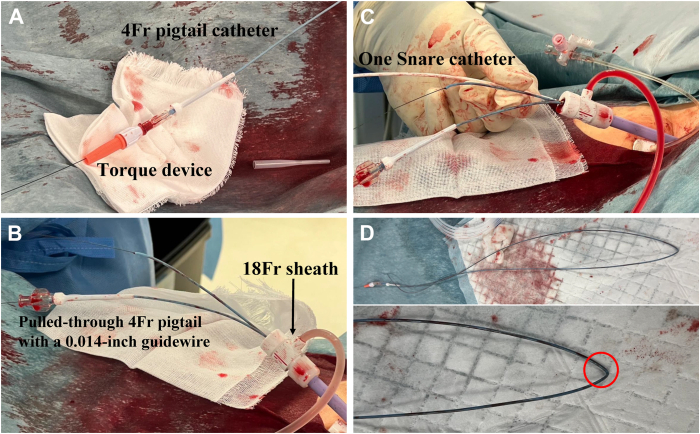
Extracorporeal component of the Wire In Pigtail cathEteR ThRoUgh Snare Twice technique. **A:** After inserting a torque device into the 0.014-in guidewire, it was secured to the proximal end of the 4-F pigtail catheter, integrating both components. **B:** The 0.014-in guidewire, along with the 4-F pigtail catheter, was externalized within the 18-F sheath. **C:** The ONE Snare catheter was opened externally, and both ends of the 0.014-in guidewire within the 4-F pigtail catheter were passed through the snare, which was then closed and reintroduced into the 18-F sheath. **D:** Image showing the 4-F pigtail catheter, containing a 0.014-in guidewire (the bottom half of the panel is an enlarged view of the top half). The guidewire remains within the pigtail catheter, even after the snare secures the lead, ensuring maintained flexibility and preventing kinking (*red circle*).

First, an 18-F sheath (Check-Flo, 40 cm, Cook Medical, Bloomington, IN) was introduced into the right femoral vein. A 4-F, 120-cm pigtail catheter (Terumo, Tokyo, Japan) was then advanced through the sheath into the RA, and the RV lead was hooked under multidirectional fluoroscopic guidance (Figure 1A). Subsequently, a 0.014-in guidewire (Hi-Torque Command, 300 cm, Abbott Vascular, Santa Clara, CA) was inserted and advanced through the pigtail catheter into the inferior vena cava. A 6-F snare catheter with a 35-mm loop (ONE Snare, Merit Medical, South Jordan, UT) was introduced alongside the pigtail catheter through the 18-F sheath. The distal end of the 0.014-in guidewire was advanced through the ONE Snare (Figure 1B), after which the snare was tightened to secure the 0.014-in guidewire. After inserting a torque device into the 0.014-in guidewire, it was then attached to the proximal end of the 4-F pigtail catheter (Figure 2A). The ONE Snare catheter grasping the 0.014-in guidewire was retracted into the 18-F femoral sheath (Figure 1C), which enabled externalization of the 0.014-in guidewire and 4-F pigtail catheter (Figure 2B).

The ONE Snare catheter was opened outside the body, and both ends of the 0.014-in guidewire within the 4-F pigtail catheter were threaded through the snare. The snare was closed and reintroduced into the 18-F sheath (Figure 2C). By simultaneously advancing and tightening the snare while applying tension to the externalized 4-F pigtail catheter, the lead was securely grasped (Figure 1D). With the WIPER TRUST technique successfully established via the femoral approach, we proceeded with adhesion dissection using a 14-F GlideLight laser sheath (Philips) through the superior approach. However, further advancement was impeded by lead-to-lead adhesion at the innominate vein. Therefore, we exchanged the sheath for an 11.5-F mechanical sheath (Byrd Dilator Sheath, Cook Medical) and proceeded with removing adhesion (Figure 1E). This dislodgment occurred because of lead-to-lead adhesion, not because of the WIPER TRUST technique. However, the distal tip of the RA lead became dislodged from the RA appendage, necessitating its extraction (Supplemental Video 1). The ONE Snare catheter was temporarily withdrawn, leaving only the pigtail catheter in place. The ONE Snare catheter was then reintroduced into the 18-F sheath and maneuvered to capture the free end of the RA lead (Figure 1F). To facilitate RA lead extraction, traction was applied to the proximal (superior approach) and distal (femoral approach) sides of the RV lead. The RA lead was then extracted using the 14-F and 16-F GlideLight laser sheaths (Figure 1G and 1H).

The procedure was resumed for RV lead removal. The ONE Snare catheter was withdrawn and opened outside the body, allowing the 0.014-in guidewire in the 4-F pigtail catheter to be threaded through the snare. This ensured secure grasping of the RV lead, facilitating the tandem approach. The 16-F GlideLight laser sheath was advanced to the grasping point (Figure 1I). The pigtail catheter was then advanced upward (Figure 1J). Countertraction was applied using the 16-F GlideLight laser sheath, allowing extraction of the RV lead via superior access (Figure 1K). Subsequently, a new RV lead (Select Secure, Medtronic, Minneapolis, MN) and a new RA lead (CapSureFix NOVUS, Medtronic) were implanted and connected to a new generator (Azure XT DR, Medtronic) (Figure 1L). The procedure was completed without any complications.

## Discussion

**Figure 3:**
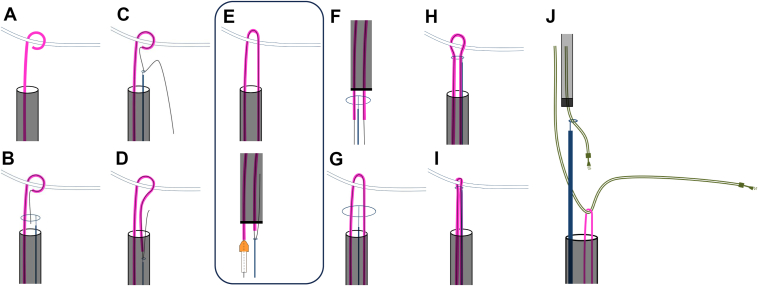
Schematic drawing of the Wire In Pigtail cathEteR ThRoUgh Snare Twice (WIPER TRUST) technique. **A:** A 4-F pigtail catheter hooks the targeted lead (see Figure 1A). **B:** A 0.014-in guidewire is inserted into the pigtail catheter, crosses over the lead, and is then threaded through the snare, which is inserted into the same sheath (see Figure 1B). **C–E:** The ONE Snare catheter, firmly grasping the 0.014-in guidewire, is retracted into the 18-F femoral sheath, externalizing the guidewire along with the 4-F pigtail catheter, which is fixed using a torque device (see Figure 1, Figure 2). **F:** The ONE Snare catheter is opened outside the body, and both ends of the 0.014-in guidewire within the 4-F pigtail catheter are passed through the snare (see Figure 2C). **G–I:** The target lead is securely grasped by advancing and tightening the snare while applying tension to the externalized 4-F pigtail catheter, successfully applying the WIPER TRUST technique via the femoral approach (see Figure 1D). **J:** During right atrial lead extraction, the ONE Snare catheter is used to secure its free end. Traction is applied to the proximal right ventricular lead via the superior approach and the distal side via the femoral approach using the externalized pigtail catheter, optimizing adhesion release and facilitating lead extraction.

Using a combined superior and femoral (tandem) approach in TLE increases the likelihood of complete procedural success.^2^^,^^5^ This approach provides geometric advantages and is associated with a reduced risk of superior vena cava injury.^6^ The needle’s eye snare is commonly used for grasping leads with inaccessible ends via the femoral approach,^7^^,^^8^ while the pigtail catheter can be more intuitive for a broader range of operators. While the Wire TRUST technique (schematically illustrated in Figure 3A–3I) relies solely on a 0.014-in guidewire, the WIPER TRUST technique incorporates a 4-F pigtail catheter in conjunction with a 0.014-in guidewire, enhancing procedural control and stability. A 0.014-in guidewire is chosen since inserting a 0.035-in guidewire into the pigtail catheter causes the pigtail portion to stretch and may dislodge the hook of the ventricular lead.^3^ The 4-F pigtail catheter provides a larger diameter, reducing the likelihood of insulation embedding and making it gentler on the lead. Furthermore, the WIPER TRUST technique eliminates the issue that the 0.014-in guidewire may bend and stiffen by incorporating a 4-F pigtail catheter. The guidewire inside the pigtail catheter maintains its flexibility, and it does not kink (Figure 2D).

The Wire TRUST technique requires a minimum 10-F sheath to accommodate simultaneous insertion of a 4-F pigtail and a 6-F snare catheter. In contrast, the WIPER TRUST technique necessitates a sheath of at least 14 F to allow pull-through of the 4-F pigtail catheter alongside the 6-F snare catheter. Given that tandem approach cases often involve prolonged lead dwell time and increased procedural complexity, making femoral extraction more likely, a sheath of ≥14 F is generally preferred, regardless of whether the WIPER TRUST technique is used. Additionally, WIPER TRUST requires pull-through of a 120-cm, 4-F pigtail catheter; thus, the sheath should be <60 cm in length (or preferably <50 cm for added flexibility). In this case, an 18-F, 40-cm sheath was used. Advancing the long sheath over the looped system may be feasible, but the snare catheter allows for flexible positioning to grip the target lead at an optimal point, differing from wire-loop techniques.^9^ Further, it may be possible to advance the pigtail catheter slightly over the target lead, though pulling the 0.014-in guidewire directly without the catheter may pose a risk of wire damage.

The key distinction from previous reports of the Wire TRUST technique is the involvement of TLE, which requires the removal of multiple leads.^3^^,^^4^ Grasping a second lead while maintaining control of the first can be technically and economically challenging. However, when the second lead has an accessible free end, as in this case, conventional snares such as the ONE Snare may provide more effective lead capture. In this case, after using the ONE Snare catheter to secure the RV lead, this catheter was removed while keeping the pull-through 0.014-in guidewire inside the pigtail catheter, allowing the same snare catheter to be reused for grasping the RA lead. Furthermore, in this case, traction was applied to the proximal end of the RV lead (via the superior approach) and the distal end (via the femoral approach using the pigtail catheter after externalization) to facilitate extraction of the RA lead (Figure 1, Figure 3 and Figure 1, Figure 3). This maneuver optimized conditions for disrupting lead-to-lead adhesions. Because traction was applied using the 4-F pigtail catheter instead of the 0.014-in guidewire, the risk of the guidewire becoming embedded in the insulation of the RV lead was considerably reduced. One advantage of the WIPER TRUST technique is that if the RV lead needs to be regrasped, the externalized ends of the pull-through pigtail catheter can be threaded through the snare, allowing the RV lead to be easily secured again without additional cost.

As long as the 4-F pigtail catheter is successfully pulled through across the lead, the RA and RV leads can be alternately secured with greater ease. Therefore, lead extractors need to be well-versed in the technique described here to enhance the safety and efficacy of TLE.

## Conclusion

The WIPER TRUST technique represents an evolution of the Wire TRUST technique and provides a safer, more effective method for grasping leads without free ends during TLE. By incorporating a 4-F pigtail catheter alongside a 0.014-in guidewire, this approach minimizes the risk of lead insulation damage and enhances procedural stability.

## Disclosures

The authors have no conflicts of interest to disclose.

## Appendix Supplementary data

Supplemental Video 1

Supplementary Video legend To facilitate lead-to-lead adhesion dissection, an 11.5Fr mechanical sheath was used, but the distal tip of the right atrial lead dislodged from the right atrial appendage during the procedure.
