# Supplementary material for: Wire In Pigtail cathEteR ThRoUgh Snare Twice (WIPER TRUST) technique for grasping leads without free ends in transvenous lead extraction
Source: HeartRhythm Case Rep. 2025 Jul 11;11(10):996–1000. doi: 10.1016/j.hrcr.2025.07.005 (PMC12666931; doi:10.1016/j.hrcr.2025.07.005)
Supplement: Supplementary Video legend — To facilitate lead-to-lead adhesion dissection, an 11.5Fr mechanical sheath was used, but the distal tip of the right atrial lead dislodged from the right atrial appendage during the procedure. [file mmc2.docx]

**Supplementary Video legends**

**Supplementary Video.**

To facilitate lead-to-lead adhesion dissection, an 11.5Fr mechanical sheath was used, but the distal tip of the right atrial lead dislodged from the right atrial appendage during the procedure.
